# Tannic acid-chelated zinc supplementation alleviates intestinal injury in piglets challenged by porcine epidemic diarrhea virus

**DOI:** 10.3389/fvets.2022.1033022

**Published:** 2022-10-10

**Authors:** Zhengfan Zhang, Sitian Wang, Liyun Zheng, Yongqing Hou, Shuangshuang Guo, Lei Wang, Liangyun Zhu, Cuifang Deng, Tao Wu, Dan Yi, Binying Ding

**Affiliations:** Hubei Key Laboratory of Animal Nutrition and Feed Science, Wuhan Polytechnic University, Wuhan, China

**Keywords:** antioxidant capacity, intestinal functions, piglets, porcine epidemic diarrhea virus, tannic acid-chelated zinc

## Abstract

Porcine epidemic diarrhea virus (PEDV) has become a challenging problem in pig industry all over the world, causing significant profit losses. Tannins and organic zinc have been shown to exert protective effects on the intestinal dysfunction caused by endotoxins. However, there is little information on tannic acid-chelated zinc (TAZ) supplementation in the diet of newborn piglets. This study was conducted to determine the effects of TAZ on the intestinal function of piglets infected with PEDV. Thirty-two 7-day-old piglets were randomly allocated to 1 of 4 treatments in a 2 × 2 factorial design consisting of 2 diets (0 or 50 mg/kg BW TAZ) and challenge (saline or PEDV). On day 9 of the trial, 8 pigs per treatment received either sterile saline or PEDV solution at 10^6^ TCID_50_ (50% tissue culture infectious dose) per pig. Pigs infected with PEDV had greater diarrhea rate and lower average daily gain (ADG) (*P* < 0.05). PEDV infection decreased plasma D-xylose concentration, most antioxidative enzyme activities in plasma and intestine, as well as the small intestinal villus height (*P* < 0.05). Plasma diamine oxidase and blood parameters were also affected by PEDV infection. Dietary supplementation with TAZ could ameliorate the PEDV-induced changes in all measured variables (*P* < 0.05). Moreover, TAZ decreased the concentration of malondialdehyde in plasma, duodenum, jejunum, and colon (*P* < 0.05). Collectively, our results indicated that dietary TAZ could alleviate PEDV induced damage on intestinal mucosa and antioxidative capacity, and improve the absorptive function and growth in piglets. Therefore, our novel findings also suggest that TAZ, as a new feed additive for neonatal and weaning piglets, has the potential to be an alternative to ZnO.

## Introduction

Porcine epidemic diarrhea virus (PEDV) spreads through feed and fecal oral route, which is a main pathogen that causes enteric diseases in swine industry (1). The virus induces apoptosis and necrosis of intestinal epithelium, mainly in the jejunum and ileum, which causes watery malabsorptive diarrhea, vomiting, and high mortality in pigs at all ages, especially during the neonatal and weanling periods (2–5). It has been demonstrated that feed additives, such as organic acids (6), organic trace minerals, medium-chain fatty acids (7), plant extracts (8), amino acid derivates (9) could ameliorate PEDV-infected intestinal injury. However, the mechanism of functional feed additives for prevention and treatment of PEDV-infected intestinal are still lacking.

In current practices, dietary supplementation with pharmacological zinc oxide (ZnO, 1600–2500 mg/kg zinc) in piglets during the first 2 weeks after weaning could prevent diarrhea (10, 11). A previous study conducted by our research team also reported that 100 mg/kg BW ZnO could improve growth performance, intestinal function, and antioxidant capacity in PEDV-infected piglets (12). However, medicinal ZnO in pig production will be disused by 2022 in Europe because of the environmental pollution and antibiotic-resistant issue (13). Therefore, hydrolyzed tannins have been widely used in piglet diets to decrease diarrhea rate, modulate intestinal health, and enhance growth performance (11, 14–16).

Studies have shown that tannic acid has various biological functions such as antioxidative, antibacterial, and antiviral property (17). However, there is still controversy on the effect of tannins in piglets (14, 18). Therefore, in the present study, we evaluated the effect of a new form of organic zinc, which is chelated with tannic acid, on the growth, antioxidative status, intestinal morphology in PEDV-infected piglets. Our findings are expected to explore an alternative to ZnO, and determine the mechanism of tannic acid-chelated zinc (TAZ) in alleviating the negative effects of PEDV in neonatal piglets.

## Materials and methods

### Animal care and diets

All animal procedures used in this study were approved by the Institutional Animal Care and Use Committee of Wuhan Polytechnic University (Number: 20161121). A total of 32 healthy 7-day-old piglets (Duroc × Landrace × Yorkshire, BW = 2.46 ± 0.21 kg) were used in this experiment. Pigs were housed individually with strict control of cross-infection in two environment-controlled nursery rooms (30 ± 2°C) and given *ad libitum* access to water throughout the study. The TAZ was obtained from the Animal Nutrition and Intestinal Health Research Group of Wuhan Polytechnic University, which contained ≥80% tannin, 6–7% zinc, crude fiber <2.00%, ash <2.50%, and moisture <8.00%. Piglets were provided a basal diet (liquid milk replacer), which was formulated to meet or exceed the nutrient requirements of suckling piglets. The milk replacer was purchased from Wuhan Anyou Feed Co., Ltd (Wuhan, China). Before feeding, the milk replacer was dissolved in warm water (45–55°C) to form a liquid feed (dry matter content of 20%) (9). Pigs were fed the liquid feed every 3 h between 8:00 am and 8:00 pm.

### Experiment design

Pigs were fed the control liquid diet or TAZ-supplemented liquid diet for 9 days before the PEDV challenge (16 pigs per group). Immediately after PEDV challenge, pigs were divided into four treatments in a 2 × 2 factorial design. The main factors consisted of diet (0 or 50 mg/kg BW TAZ supplementation in diet; +TAZ or –TAZ) and challenge (PEDV or saline administration; +PEDV or –PEDV). On day 9 of the experiment, eight pigs in each dietary treatment were orally administered with either PEDV at a dose of 10^6^ TCID_50_ (50% tissue culture infectious dose) per pig or the same volume of sterile saline (Control). On day 12 of the trial, 10% D-xylose (1 mL/kg BW) was orally administrated to piglets to determine the intestinal absorption capacity and mucosal integrity (9). One hour later, all piglets were weighed and blood samples were collected from the anterior vena cava, and then all pigs were sacrificed under sodium pentobarbital anesthesia (50 mg/kg BW, iv) to obtain intestinal samples (12).

### Collection of blood and intestinal samples

As mentioned previously, all blood samples were collected from anterior vena cava of piglets into heparinized vacuum tubes (Becton-Dickinson Vacutainer System, Franklin Lake, NJ, USA) at 1 h post D-xylose administration on day 12 of the trial (19). Blood samples were centrifuged at 3000 rpm for 15 min at 4°C to obtain plasma, which was then stored at −20°C until analysis.

After slaughtering, the pig abdomen was opened immediately and the whole gastrointestinal tract was exposed. The intestine was dissected free of the mesentery and placed on a chilled stainless steel tray. The 1- and 10-cm segments were obtained from the distal duodenum, mid-jejunum, mid-ileum and mid-colon, respectively (19, 20). The 5 cm intestinal segments were flushed gently with ice-cold phosphate buffered saline (PBS, pH = 7.4) and then placed in 4% fresh, chilled formalin solution for histological measurements. The 10-cm segments were opened longitudinally and the contents were flushed with ice-cold PBS. Mucosa was collected by scraping using a sterile glass microscope slide at 4°C, rapidly frozen in liquid nitrogen, and stored at −80°C until analysis. All samples were collected within 15 min after killing.

### Growth performance and diarrhea rate

Piglets were weighted on d 0, 9, and 12 of the experiment to calculate the average daily gain (ADG). Health status and diarrhea score were recorded throughout the experimental period. The fecal score was classified into four levels: 0 = strip or granular feces, 1 = soft stool feces, 2 = thick and water feces, and 3 = water feces. Score ≥ 2 was considered diarrhea. The formula of diarrhea rate was given as follows: diarrhea rate (DR) = total number of pigs with diarrhea/(total number of test piglets × test days) × 100% (21).

### Blood parameters

The concentrations of blood biochemical parameters, such as total protein (TP), albumin (ALB), aspartate transaminase (AST), alanine transaminase (ALT), γ-glutamyltransferase (GGT), alkaline phosphatase (ALP), lactate dehydrogenase (LDH), total cholesterol (TC), triacylglycerol (TG), glucose (GLU), calcium (Ca), phosphorus (P), creatinine (CREA), high density lipoprotein (HDL), and low density lipoprotein (LDL) were measured with Wako kits (Wako Pure Chemical Industries, Ltd., Osaka, Japan) using a Hitachi 7060 Automatic Biochemical Analyzer (Hitachi, Tokyo, Japan).

### Determination of D-xylose and diamine oxidase activity in plasma

Plasma D-xylose concentration and DAO activity were determined by colorimetric method using commercial kits (Nanjing Jiancheng Bioengineering Institute, Nanjing, China). All assays were performed according to the instructions of manufacturer.

### Antioxidant capacity in plasma and intestinal mucosa

Plasma, mucosa of duodenum, jejunum, ileum, and colon were used for analysis of antioxidative enzymes and related products. The activities of glutathione peroxidase (GSH-Px), catalase (CAT), total superoxide dismutase (T-SOD), total antioxidant capacity (T-AOC), as well as the concentration of hydrogen peroxide (H_2_O_2_) and malondialdehyde (MDA) were determined by using commercially available kits (Nanjing Jiancheng Bioengineering Institute, Nanjing, China) according to the protocols of manufacturer (22). Assays were performed in triplicate.

### Intestinal histomorphology

Intestinal histomorphology were examined according to the method of Yi et al. (21). Briefly, the fixed intestinal segments were embedded in paraffin. Consecutive 5 μm sections were cut and then stained with haematoxylin and eosin. Intestinal morphology was determined using a light microscope (Leica Microsystems, Wetzlar, Germany) with Leica Application Suite image analysis software (Leica Microsystems, Wetzlar, Germany). The villus height, villus width at half-height, and crypt depth were measured from 10 randomly selected villi and associated crypts on each section at 40 × magnification. Villus height was measured from the tip of villus to the crypt opening and crypt depth was measured from the base of crypt to the level of crypt opening. The villus height/crypt depth ratio and villous surface area were then calculated from these measurements.

### Statistical analyses

All data were analyzed by one-way ANOVA using the GLM procedure of SPSS 20.0 software appropriate for a 2 × 2 factorial design (SPSS Inc. Chicago, IL, USA). The statistical model consisted of the effects of diet (+TAZ vs. –TAZ) and challenge (saline vs. PEDV) and their interactions. Data were expressed as means and pooled SEMs. In cases where the differences were significant, the means were compared by Duncan's multiple range test. A value of *P* < 0.05 were considered significant, and 0.05 ≤ *P* < 0.10 as trends.

## Results

### Average daily gain and diarrhea rate

The effect of TAZ on ADG and diarrhea rate in PEDV-infected piglets is shown in Table 1. During days 0–9 (pre-infection), there was no difference in the ADG and DR of pigs fed the control and TAZ-supplemented diets (*P* > 0.05). During days 9–12 of the trial (post-infection), PEDV infection decreased the ADG, and increased the diarrhea rate (*P* < 0.05). There were interactive effects between TAZ and PEDV, the TAZ administration mitigated diarrhea and increased the ADG induced by PEDV infection (*P* < 0.05).

**Table 1:** The effect of tannic acid-chelated zinc on ADG and diarrhea rate in PEDV-infected piglets.

**Items**	**–PEDV**		+**PEDV**		**SEM**	***P-*** **values**		
	**–TAZ**	**+TAZ**	**–TAZ**	**+TAZ**		**PEDV**	**TAZ**	**PEDV × TAZ**
**Days 0–9**								
ADG (g)	83.5	88.6	85.6	90.6	8.65	0.845	0.762	0.895
Diarrhea rate (%)	4.3	3.5	4.8	3.2	0.98	0.345	0.421	0.598
**Days 9–12**								
ADG (g)	120.2^a^	143.4^a^	31.9^b^	112.7^a^	9.58	<0.001	<0.001	0.014
Diarrhea rate (%)	0^c^	0^c^	83.8^a^	58.3^b^	2.35	<0.001	<0.001	0.012

Values are mean and pooled SEM, n = 8. PEDV, porcine epidemic diarrhea virus; TAZ, tannic acid-chelated zinc. ^a, b, c^Within a row, means with different superscripts differ, P < 0.05. ADG, average daily gain.

### Blood parameters

The effect of TAZ on blood parameters in PEDV-infected piglets is shown in Table 2. Compared with non-infected pigs, PEDV-infected piglets had lower concentrations of ALP, TC, P, HDL, LDL, and LDH in plasma, and had greater concentration of ALT, TG and GLU (*P* < 0.05). Pigs fed the TAZ diet had a lower plasma ALT level, and tended to have greater plasma GGT than the control pigs (*P* = 0.090). There were interactive effects between PEDV and TAZ on plasma ALB and P concentrations (*P* < 0.05). The concentrations of ALB and P was increased in pigs infected with PEDV fed the TAZ diet compared with the PEDV-infected pigs fed a diet without TAZ (–TAZ) (*P* < 0.05), whereas there was no difference in these parameters in saline (–PEDV) treatments (*P* > 0.05).

**Table 2:** The effect of tannic acid-chelated zinc on blood parameters in PEDV-infected piglets.

**Items**	**–PEDV**		+**PEDV**		**SEM**	***P*** **-values**		
	**–TAZ**	**+TAZ**	**–TAZ**	**+TAZ**		**PEDV**	**TAZ**	**PEDV × TAZ**
TP (g/L)	6.02	6.17	6.19	6.29	0.104	0.520	0.579	0.895
ALB (g/L)	2.96^ab^	2.74^b^	2.70^b^	3.13^a^	0.056	0.142	0.937	0.048
AST (U/L)	38.83	37.17	32.30	35.29	1.761	0.262	0.859	0.532
ALT (U/L)	54.83	53.33	65.40	55.14	1.574	0.009	0.019	0.157
ALP (U/L)	780.17	865.33	625.70	546.71	42.385	0.005	0.968	0.299
TC (mg/dL)	201.62	241.42	107.25	106.40	13.233	<0.001	0.238	0.219
TG (mg/dL)	40.49	27.05	60.83	47.25	4.371	0.017	0.102	0.993
GLU (mg/dL)	73.45	80.03	90.03	92.57	3.539	0.049	0.522	0.776
Ca (mg/dL)	10.62	10.53	11.23	22.95	3.027	0.303	0.357	0.350
P (mg/dL)	9.60^a^	9.15^ab^	7.89^c^	8.67^b^	0.161	<0.001	0.471	0.011
CREA (mg/dL)	0.972	0.755	0.635	0.779	0.074	0.310	0.811	0.245
HDL (mg/dL)	99.89	120.95	48.57	45.12	6.966	<0.001	0.254	0.116
LDL (mg/dL)	155.22	196.78	67.53	62.79	13.547	<0.001	0.324	0.217
GGT (U/L)	38.17	39.00	30.60	45.00	4.253	0.858	0.090	0.129
LDH (U/L)	832.03	864.70	728.01	752.94	18.746	<0.001	0.398	0.909

Values are mean and pooled SEM, n = 8. PEDV, porcine epidemic diarrhea virus; TAZ, tannic acid-chelated zinc. ^a, b, c^Within a row, means with different superscripts differ, P < 0.05. TB, total bilirubin; TP, total protein; ALB, albumin; AST, aspartate transaminase; ALT, alanine transaminase; GGT, γ-glutamyltransferase; ALP, alkaline phosphatase; LDH, lactate dehydrogenase; TC, total cholesterol; TG, triacylglycerol; GLU, glucose; Ca, calcium; P, phosphorus; CREA, creatinine; HDL, high density lipoprotein; LDL, low density lipoprotein.

### Diamine oxidase activity and D-xylose concentration

Data on plasma DAO activity and D-xylose concentration are summarized in Table 3. The PEDV-infected pigs had greater activity of DAO and lower D-xylose concentration in plasma than non-infected pigs (*P* < 0.05). Pigs fed the TAZ diet showed lower plasma DAO activity and greater D-xylose concentration than pigs in control group (*P* < 0.05).

**Table 3:** The effect of tannic acid-chelated zinc on DAO activity and D-xylose concentration in PEDV-infected piglets.

**Items**	**–PEDV**		+**PEDV**		**SEM**	***P*** **-values**		
	**–TAZ**	**+TAZ**	**–TAZ**	**+TAZ**		**PEDV**	**TAZ**	**PEDV × TAZ**
DAO (U/L)	18.86	18.28	22.58	19.78	0.466	0.001	0.021	0.115
D-xylose (mmol/L)	3.23	3.63	1.83	2.08	0.141	<0.001	<0.001	0.293

Values are mean and pooled SEM, n = 8. PEDV, porcine epidemic diarrhea virus; TAZ, tannic acid-chelated zinc; DAO, Diamine oxidase.

### Plasma antioxidant capacity

The effect of TAZ on plasma antioxidant capacity in PEDV-infected piglets is shown in Table 4. Compared with non-infected pigs, PEDV-infected pigs had lower GSH-Px and T-SOD activity in plasma, and greater H_2_O_2_ concentration than those in the control treatment (*P* < 0.05). The activity of CAT in plasma was increased in TAZ, and the plasma H_2_O_2_ and MDA concentration was decreased compared with the control group (*P* < 0.05). There was PEDV × TAZ interaction on the plasma CAT activity (*P* < 0.05). The data showed that TAZ supplementation was more effective to increase the activity of CAT in plasma of PEDV-infected pigs than non-infected pigs (*P* < 0.05). However, the T-AOC in plasma was not affected either by PEDV or dietary TAZ (*P* > 0.05).

**Table 4:** The effect of tannic acid-chelated zinc on plasma antioxidant capacity in PEDV-infected piglets.

**Items**	**–PEDV**		+**PEDV**		**SEM**	***P*** **-values**		
	**–TAZ**	**+TAZ**	**–TAZ**	**+TAZ**		**PEDV**	**TAZ**	**PEDV × TAZ**
T-AOC (mM)	3.077	2.902	3.315	2.913	0.102	0.550	0.176	0.588
GSH-Px (U/ml)	363.9	370.1	329.1	359.4	5.95	0.045	0.102	0.273
T-SOD (U/ml)	86.33	86.69	80.74	82.25	0.923	0.006	0.567	0.727
CAT (U/ml)	3.68^c^	3.99^b^	3.09^d^	5.03^a^	0.151	0.006	<0.001	<0.001
H_2_O_2_ (nmol/L)	67.25	63.05	95.43	79.66	5.98	0.001	0.012	0.251
MDA (nmol/mL)	0.255	0.215	0.262	0.210	0.007	0.938	<0.001	0.587

Values are mean and pooled SEM, n = 8. PEDV, porcine epidemic diarrhea virus; TAZ, tannic acid-chelated zinc. ^a, b, c, d^Within a row, means with different superscripts differ, P < 0.05. T-AOC, total antioxidant capacity; GSH-Px, glutathione peroxidase; CAT, catalase; T-SOD, total superoxide and dismutase; H_2_O_2_, hydrogen peroxide; MDA, malondialdehyde.

### Intestinal antioxidant capacity

The effect of TAZ on the intestinal antioxidant capacity in PEDV-infected piglets is shown in Table 5. Compared with non-infected pigs, PEDV-infected pigs had lower GSH-Px and CAT activities in duodenum and jejunum, and greater MDA concentration in duodenum than those in the control treatment (*P* < 0.05). The concentration of H_2_O_2_ in colon was tended to increase in PEDV treatments compared with the non-infected pigs (*P* = 0.076). Pigs fed the TAZ diet had greater T-AOC and GSH-Px in duodenum, jejunum, and colon (*P* < 0.05), T-SOD in duodenum and jejunum (*P* < 0.05), tended to have higher CAT in jejunum (*P* = 0.075), and lower MDA concentration in duodenum, jejunum, and colon (*P* < 0.05). The colon H_2_O_2_ concentration was also decreased in the TAZ treatment compared with the control (*P* < 0.05). There were PEDV × TAZ interactions on the GSH-Px in duodenum and jejunum, jejunal T-SOD, as well as MDA and H_2_O_2_ concentration in colon (*P* < 0.05).

**Table 5:** The effect of tannic acid-chelated zinc on intestinal antioxidant capacity in PEDV-infected piglets.

**Items**	**–PEDV**		+**PEDV**		**SEM**	***P*** **-values**		
	**–TAZ**	**+TAZ**	**–TAZ**	**+TAZ**		**PEDV**	**TAZ**	**PEDV × TAZ**
**Duodenum**								
T-AOC (mmol/g protein)	0.179	0.655	0.202	0.542	0.046	0.197	<0.001	0.058
GSH-Px (U/mg protein)	31.98^a^	30.43^a^	19.96^b^	33.44^a^	1.333	<0.001	0.001	0.011
T-SOD (U/mg protein)	204.04	288.93	229.10	247.07	10.665	0.649	0.010	0.081
CAT (U/mg protein)	6.86	8.09	4.80	6.09	0.434	0.015	0.113	0.966
MDA (nmol/mg protein)	0.544	0.172	0.640	0.287	0.045	0.040	<0.001	0.839
H_2_O_2_ (μmol/g protein)	3.739	3.702	3.745	3.778	0.153	0.901	0.996	0.916
**Jejunum**								
T-AOC (mmol/g protein)	0.175	0.257	0.168	0.218	0.011	0.120	<0.001	0.267
GSH-Px (U/mg protein)	28.96^b^	46.37^a^	22.87^c^	44.18^a^	2.134	<0.001	0.013	<0.001
T-SOD (U/mg protein)	173.26^b^	357.59^a^	213.70^b^	194.31^b^	21.193	0.099	0.031	0.009
CAT (U/mg protein)	19.83	23.78	15.51	17.32	0.964	0.002	0.075	0.493
MDA (nmol/mg protein)	0.498	0.355	0.605	0.350	0.028	0.182	<0.001	0.149
H_2_O_2_ (μmol/g protein)	3.182	6.894	2.985	2.084	0.987	0.214	0.481	0.252
**Ileum**								
T-AOC (mmol/g protein)	0.977	0.980	0.984	0.981	0.006	0.739	0.990	0.782
GSH-Px (U/mg protein)	29.95	32.00	23.74	31.42	2.060	0.187	0.065	0.270
T-SOD (U/mg protein)	161.72	170.77	175.39	184.17	3.651	0.065	0.214	0.984
CAT (U/mg protein)	1.86	1.79	1.91	1.78	0.034	0.779	0.157	0.702
MDA (nmol/mg protein)	0.655	0.679	0.645	0.677	0.019	0.892	0.498	0.914
H_2_O_2_ (μmol/g protein)	0.843	0.969	0.875	1.002	0.068	0.820	0.384	0.999
**Colon**								
T-AOC (mmol/g protein)	1.071	1.086	1.091	1.112	0.005	0.012	0.044	0.715
GSH-Px (U/mg protein)	4.99	6.37	4.76	7.68	0.303	0.175	<0.001	0.058
T-SOD (U/mg protein)	216.09	212.23	223.35	226.71	6.519	0.438	0.986	0.795
CAT (U/mg protein)	3.80	4.38	3.52	3.60	0.235	0.285	0.499	0.610
MDA (nmol/mg protein)	1.389^a^	1.398^a^	1.404^a^	0.966^b^	0.062	0.055	0.049	0.041
H_2_O_2_ (μmol/g protein)	1.151^b^	1.210^b^	1.552^a^	1.045^b^	0.049	0.076	0.002	<0.001

Values are mean and pooled SEM, n = 8. PEDV, porcine epidemic diarrhea virus; TAZ, tannic acid-chelated zinc. ^a, b^Within a row, means with different superscripts differ, P < 0.05. T-AOC, total antioxidant capacity; GSH-Px, glutathione peroxidase; CAT, catalase; T-SOD, total superoxide and dismutase; H_2_O_2_, hydrogen peroxide; MDA, malondialdehyde.

### Intestinal morphology

Data on the small intestinal histomorphology are summarized in Table 6. PEDV infection decreased villus height, villus height/crypt depth ratio, and villous surface area in all small intestinal segments (*P* < 0.05), and increased the crypt depth in small intestine and colon (*P* < 0.05). There were PEDV × TAZ interactions in villus height in the jejunum and villus height/crypt depth ratio in duodenum and jejunum, as well as the crypt depth in the small intestine and colon (*P* < 0.05). Data indicated that TAZ supplementation could increase the duodenal and jejunal villus height, villus height/crypt depth ratio and villous surface area, and decrease the crypt depth in duodenum, jejunum and colon (*P* < 0.05), as compared to the control (–TAZ).

**Table 6:** The effect of tannic acid-chelated zinc on the intestinal morphology in PEDV-infected piglets.

**Items**	**–PEDV**		+**PEDV**		**SEM**	***P*** **-values**		
	**–TAZ**	**+TAZ**	**–TAZ**	**+TAZ**		**PEDV**	**TAZ**	**PEDV × TAZ**
**Villus height (μm)**								
Duodenum	205.4	311.4	141.5	196.6	15.27	<0.001	0.001	0.198
Jejunum	198.2^b^	274.2^a^	75.9^c^	94.2^c^	17.90	<0.001	0.001	0.020
Ileum	183.6	188.1	93.8	104.2	9.68	<0.001	0.307	0.687
**Crypt depth (μm)**								
Duodenum	80.6^b^	90.1^b^	147.4^a^	98.8^b^	6.64	0.040	0.001	0.007
Jejunum	98.4^b^	95.9^b^	135.7^a^	46.8^c^	7.99	<0.001	0.004	0.001
Ileum	91.0	94.1	155.5	154.9	4.27	<0.001	0.734	0.623
Colon	180.38^b^	178.57^b^	244.23^a^	188.92^b^	7.021	<0.001	0.020	0.030
**Villus height/Crypt depth**								
Duodenum	2.548^a^	3.456^b^	0.960^c^	1.990^bc^	0.060	<0.001	<0.001	0.024
Jejunum	2.014^a^	2.859^a^	0.559^b^	2.013^a^	0.038	0.023	0.026	0.013
Ileum	2.018	1.999	0.603	0.673	0.025	<0.001	0.112	0.557
**Villous surface area (μm** ^**2**^ **)**								
Duodenum	37635.7	49987.4	17689.6	26744.8	2975.00	<0.001	0.011	0.660
Jejunum	23158.1	38531.7	20353.7	24418.4	2583.74	0.004	0.010	0.557
Ileum	25161.3	24670.8	12298.0	13853.4	1424.92	<0.001	0.727	0.504

Values are mean and pooled SEM, n = 8. PEDV, porcine epidemic diarrhea virus; TAZ, tannic acid-chelated zinc. ^a, b, c^Within a row, means with different superscripts differ, P < 0.05.

## Discussion

In the last decades, PEDV outbreaks all over the world induced huge economic loses in swine industry (23). Although a series of feed additive were evaluated to prevent PEDV, the results were inconsistent. As a potential alternative to inorganic ZnO to alleviate diarrhea, we set up a PEDV infection model to investigate the protective effect of TAZ on growth, antioxidant capacity and intestinal morphology in piglets.

In the present study, infected pigs exhibited the symptoms of PEDV, such as diarrhea, vomiting and thin intestinal wall. PEDV infection decreased the ADG, and increased the diarrhea rate of piglets, which was consistent with previous studies (24–26). Our previous studies also showed that oral administration of 10^4.5^ TCID_50_ resulted in retarded growth and sever diarrhea in piglets (9, 12). Dietary TAZ alleviated the ADG reduction caused by infection. These results may be related to the interference effect on the integrity of enveloped structure of PEDV by tannin and zinc, and inhibition of the reproduction of pathogens (27). In agreement with our studies, lots of studies also showed that tannin and zinc (ZnO and organic zinc) improved the growth performance of piglets (11, 14–16). In addition, dietary administration of ZnO decreased the fecal score in a previous study, thus alleviating the ADG reduction caused by PEDV (12). These results indicated that TAZ could be a potential substitute of ZnO to prevent diarrhea and promote growth in neonatal and weaning piglets.

In our study, we found that PEDV increased the concentrations of ALT, TG and GLU in plasma, which indicated that PEDV infection already resulted in inflammatory reaction in piglets. It was reported that injury of gastrointestinal tract and liver could cause the increment of blood ALT and AST, which can sensitively reflect the function of the liver (28, 29). In this study, it was observed that the TAZ groups reduced plasma ALT, indicating that TAZ supplementation may have therapeutic effects on the hepatic architecture and function damage induced by PEDV infection (9). Moreover, the blood P concentration was increased in pigs infected with PEDV fed the TAZ diet, which may indicate that TAZ improve the integrity of intestinal epithelium, leading higher nutrient digestibility.

Plasma DAO activity and D-xylose can be used as indicators for the integrity of intestinal barrier, which is the basis for preventing pathogenic bacteria, virus, and other harmful substances (30, 31). Impaired intestine is a major cause of diarrhea, and the concentration of D-xylose in blood and urine will decrease because of malabsorption, and the activity of DAO will increase after the damage of the intestine mucosa (9, 12, 32). In consistent with previous studies, the plasma D-xylose content was decreased, and the DAO activity was increased after PEDV infection, indicating that PEDV induced intestinal epithelial cell apoptosis and impaired intestinal function. Interestingly, dietary supplementation of TAZ reduced the DAO activity and increased D-xylose concentration in plasma of piglets, indicating that TAZ is beneficial to reduce intestinal permeability, which also further explains the decreased diarrhea rate in the present study.

Oxidative damage of cell and tissues by weanling stress, mycotoxin, and virus is well documented (33–35). In the present study, PEDV challenge decreased plasma and intestinal mucosal GSH-Px, T-SOD, and CAT activities, while increased MDA and H_2_O_2_, indicating that PEDV successfully induced humoral and intestinal mucosal oxidative injury in piglets. Interestingly, supplementation with TAZ mitigated these series of oxidative damage. The hydroxyl groups of phenol rings are responsible for a strong antioxidant function of TAZ (36). A series of studies have reported that tannin rich diets could improve the antioxidative capacity in pigs. Furthermore, it was reported that polyphenols extracted from grape seeds, gallnut and chestnut improved the antioxidant status of pigs in challenge models (33, 34, 37). Different forms of zinc, especially the organic zinc sources were also reported to enhance the endogenous antioxidant defenses by acting on antioxidant enzymes and the synthesis of the metallothionein proteins, which are able to scavenge free radicals, such as hydroxyl radicals and reactive oxygen species (38, 39).

Intestinal health is the basis for meet the growth potential of piglets. Villus height, crypt depth and villous surface area are strongly related to the absorptive function, which are well accepted as indicators to reflect the morphological integrity of small intestine in animals (40, 41). In this study, PEDV infection decreased villus height and villus height/crypt depth ratios, and increased crypt depth in all segments of the small intestine, suggesting that PEDV induced intestinal structural damage and increased mucosal permeability, which was in agreement with our previous studies (9, 12). Notably, we found that TAZ supplementation increased villus height, villous surface area, and villus height/crypt depth ratios, as well as the crypt depth in colon. In consistent with our study, some studies on pigs also found that fruits and Chinese medicinal herb original tannins, and zinc could improve these intestinal morphological parameters (12, 33, 42–44). These results could also explain the better growth performance in +TAZ treatments in our study.

## Conclusion

In conclusion, we provide significant evidence for the effect of TAZ on growth performance, antioxidant capacity, intestinal morphology in piglets. Supplementation with TAZ could alleviate PEDV-induced growth retardation, oxidative stress, intestinal integrity damage in the neonatal piglets model. Our novel findings also suggest that TAZ, as a new feed additive for neonatal and weaning piglets, has the potential to be an alternative to ZnO.

## Data availability statement

The raw data supporting the conclusions of this article will be made available by the authors, without undue reservation.

## Ethics statement

The animal study was reviewed and approved by the Institutional Animal Care and Use Committee of Wuhan Polytechnic University (Number: 20161121).

## Author contributions

ZZ analyzed the data and wrote the manuscript. SW and LZheng conducted the animal experiment and analyzed the data. SG read and revised the manuscript. LZhu, CD, TW, and DY also performed the experiment work. YH and BD designed the study and acquired funding. All authors contributed to the article and approved the submitted version of the manuscript.

## Funding

This research was financially supported by the Hubei Provincial Central Leading Local Science and Technology Development Fund Project (No. 2021BGE047).

## Conflict of interest

The authors declare that the research was conducted in the absence of any commercial or financial relationships that could be construed as a potential conflict of interest.

## Publisher's note

All claims expressed in this article are solely those of the authors and do not necessarily represent those of their affiliated organizations, or those of the publisher, the editors and the reviewers. Any product that may be evaluated in this article, or claim that may be made by its manufacturer, is not guaranteed or endorsed by the publisher.
